# Mid-infrared hyperspectral sensor based on MEMS Fabry-Pérot interferometer for stand-off sensing applications

**DOI:** 10.1038/s41598-022-23758-w

**Published:** 2022-11-12

**Authors:** Abba Saleh, Mikhail Mekhrengin, Timo Dönsberg, Teemu Kääriäinen, Guillaume Genoud, Juha Toivonen

**Affiliations:** 1 Photonics Laboratory, Tampere University, Tampere, 33101 Finland; 2 VTT Technical Research Centre of Finland Ltd, Espoo, 02150 Finland

**Keywords:** Optics and photonics, Applied optics, Optical techniques, Other photonics

## Abstract

We report a novel hyperspectral sensor employing a Fabry-Pérot interferometer based on micro-electro-mechnical system and a custom mid-infrared supercontinuum laser. The Fabry-Pérot interferometer allows on-axis filtering, of spectral components of supercontinuum light backscattered from a target, with a spectral resolution of about 25 nm. We demonstrated hyperspectral identification of black polypropylene (PP) and polyethylene (PE500) using the 3–3.5 $\mu$m region of the supercontinuum spectrum and a corresponding measurement rate of 62.5 spectra / s. The resulted spectra show excellent agreement with the reference based on an FTIR spectrometer. Furthermore, we showed that the coloring of the plastics has no effect on their identification at this wavelength range.

## Introduction

A hyperspectral sensor measures transmitted or backscattered light from a target, probing multiple spectral components of the light to enable analysis of the target. Hyperspectral sensors have been widely used in various applications including agriculture^1^, medicine^2^ and mineral exploration^3^. Conventional hyperspectral sensors and cameras rely on passive target illumination, typically ambient lightening, which make them prone to misinterpretation due to any fluctuations in the illumination spectrum as it undermines the integrity of the signal. However, recent advances in nonlinear fiber optics have led to the development of spatially coherent yet broadband fiber lasers, termed supercontinuum (SC) lasers^4,5^, enabling active target illumination for robust multi-spectral^6,7^ as well as hyperspectral^8–10^ sensing applications with very high signal-to-noise ratio.

Furthermore, significant progress in terms of the SC spectrum expanding into the mid-infrared (MIR), have been reported in the literature^11,12^, with significantly high average output power^13^, high repetition rate^14^ and femtosecond pulse durations^15,16^, opening the door for various applications in the MIR including spectroscopy^17^, imaging^18^ and optical coherence tomography (OCT)^19^. The MIR spectral region offers novel perspective for a more accurate analysis of a target as molecules demonstrate strong and characteristic absorption in this region, termed the molecular fingerprint, due to the strong fundamental vibrational transitions^20^. The aforementioned properties of MIR SC have opened up new possibilities for hyperspectral sensing applications in the MIR spectral region. However, a fast and robust filtering of various spectral components of the MIR SC spectrum is required to meet the demands of hyperspectral sensing applications. Some promising solutions based on a spectrometer comprising a miniaturized Fabry-Pérot interferometer have been proposed in the literature^8^. However, the integrated nature of the spectrometer limits the sensor design flexibility and photodetector choice as both the detector and the Fabry-Pérot interferometer are incorporated as a single unit. Thus, further study is required to fully realize the commercial potential of MIR hyperspectral sensors.

Herein, we present an active hyperspectral sensor (AHS) using a combination of a MEMS-based Fabry-Pérot interferometer (FPI) and a spectrally tailored SC light source covering up to 3.5 $\mu$m of the MIR spectral region. The voltage tunable FPI enables compact, cost effective and on axis non-dispersive filtering of spectral components of the SC light reflected from a target. The standalone nature of the FPI allows flexible instrument design as it can be placed at any desirable location on the instrument. We showed for the first time, to the best of our knowledge, the suitability of FPI for hyperspectral sensing of plastics. We demonstrated hyperspectral identification of black plastics as their detection is tedious in the near infrared (NIR) due to the carbon based additives which significantly absorb the NIR light. Black polyethylene (PE500) and polypropylene (PP) are specifically chosen as sample materials as they are one of the most important black plastic waste especially among waste electrical and electronic equipment (WEEE)^21^. Additionally, they have very similar absorption features which makes them very difficult to distinguish. We successfully measured their reflectance spectra using the 3 – 3.5 $\mu$m spectral band of the SC spectrum. The resulting spectra strongly correspond to their reference based on an FTIR spectrometer. The technique is further extended to detection of white plastics to validate its applicability to differently colored plastics. This emphasizes the great potential of the technique for plastic wastes sorting, in recycling processes, and other hyperspectral sensing applications.

## Methods and results

**Figure 1:**
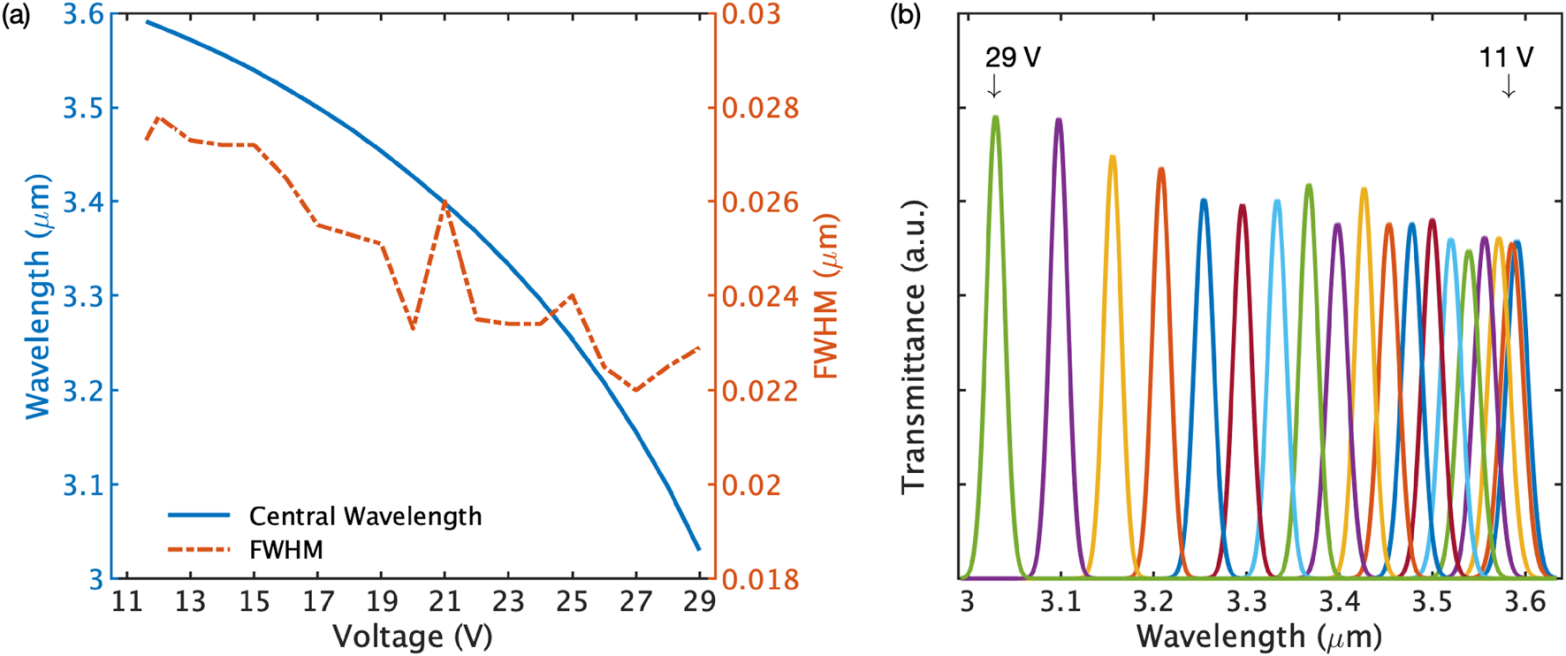
Tuning parameters of the Fabry-Pérot interferometer. (**a**) The blue line represents the corresponding central wavelength with respect to the tuning voltage, and the overlay red (dashed) line shows the full-width-half-maximum (FWHM). (**b**) Transmittance spectra of the Fabry-Pérot interferometer are shown as a function of the operating voltage in the range of 11–29 V with constant voltage increments indicated by different colors in the figure.

Operational parameters of the Fabry-Pérot interferometer are shown in Fig. 1. The miniaturized MEMS-based tunable FPI filter is part of the MEMS-FPI solutions developed at VTT Technical Research Centre of Finland Ltd^22–25^. The FPI resonator comprises two highly reflective mirrors separated by an air gap. Constructively interfered light between the mirrors is transmitted, and the transmission band is defined by the air gap between the mirrors. The air gap is tuned by applying a voltage between the mirrors and, thus, the transmission band of the FPI can be tuned to the range of interest. Figure 1a presents the tuning parameters of the FPI. The operating voltage of the FPI is in the range of 0 – 29 V which corresponds to a wavelength tuning range of 3000 – 3600 nm. The full width half maximum (FWHM) of the transmission band remains in the range of 22 – 28 nm over the whole operating range of the FPI. An example transmittance spectra of the FPI at a constant voltage increment is shown in Fig. 1b.

**Figure 2:**
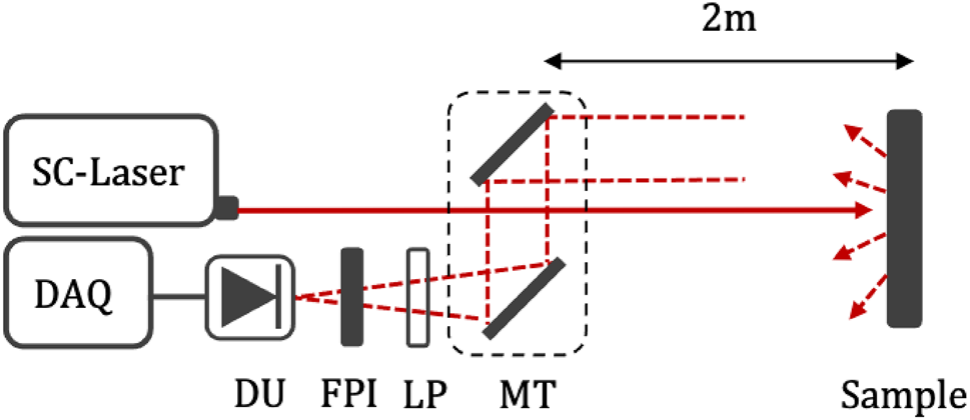
Experimental setup for stand-off hyperspectral reflectance measurement. Abbreviations: MT—mirror telescope, LP—longpass filter, DU—detection unit, FPI—Fabry-Pérot interferometer and DAQ—data acquisition.

The experimental arrangement is presented in Fig. 2. A custom SC light source^26^ producing 10 kW peak power sub-nanosecond pulses at a repetition rate of 100 kHz (more detailed description of the supercontinuum light source can be found in Ref. 26) is directed towards a black plastic target having a thickness of 3–5 mm and located at a distance of 2 m. The SC light is partially absorbed upon incidence on the target while being scattered. The backscattered light is collected via a mirror telescope arrangement (MPD399V–M01 & PFE10–P01, Thorlabs) then filtered by a longpass filter (SLWP-2989-000453,NOC) to cut out wavelengths below 3000 nm. The FPI scans across 3050–3600 nm spectral range at a frequency of 62.5 Hz. The light transmitted through the FPI is focused onto a photodetector (PV-3TE-5, VIGO Systems). The measured signal is amplified with a custom bandpass-filtered amplifier having a voltage gain of 250 and passband of 3.5 kHz - 10 MHz. The measured sub-nanosecond SC pulses are temporarily stretched due to the 10 MHz cut-off frequency of the amplifiers. They are digitized using a 14-bit analog-to-digital converter (LTC2145-14, Analog Devices, Inc.) which is a part of a development board (STEMlab 125-14, Red Pitaya d.d.). The digitized signal is processed using a system on a chip (SoC) solution (Zynq 7010, Xilinx). The SoC contains field-programmable gate array (FPGA)-based programmable logic (Artix 7, Xilinx) and a processor (ARM Cortex-A9 MPCore, Arm Holdings). The FPGA part of SoC removes constant component of the signal and integrates all data samples related to one SC pulse into one value while the processor send the integrated data values to the PC via Ethernet. The FPI voltage modulation and SoC acquisition software are synchronized using the same external trigger as applied for the SC laser. Although the FPI scans continuously during the measurement, the measured spectra seem to show discrete values as transmitted signal is sampled by FPGA with 125 MHz-sampling rate, which allows to distinguish and integrate individual SC pulses. And they are then averaged over 100-µs-long periods of time, corresponding to independent spectral channels.

The reflectance spectrum of a sample can be derived using

1 $$\begin{aligned} R = \frac{I_S-N}{I_R-N}, \end{aligned}$$

where $I_S$ and $I_R$ are the measured intensity spectrum of the sample and reference target respectively, and *N* is the background noise. As a reference target, we used a ground glass diffuser (DG10-120-M01, Thorlabs) having spectrally flat reflectance of 97.5 $\%$ over the probed wavelength range.

**Figure 3:**
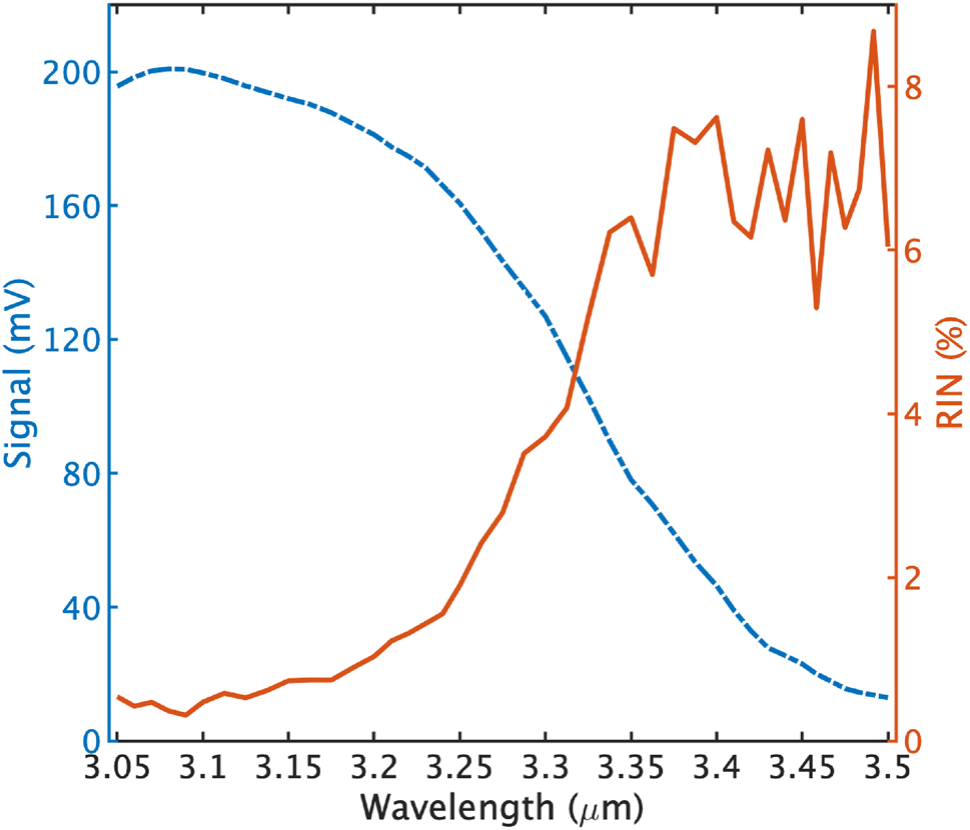
Measured backscattered SC spectrum (– -) from a diffuser and the corresponding relative intensity noise (—) based on ten consecutive measurements.

Figure 3 shows a measured backscattered SC spectrum from the diffuser and the corresponding relative intensity noise (RIN) based on 10 different measurements. The resulted spectral shape is mainly due to the spectrum of the SC light source, as the diffuser and detector responses are flat over the probed wavelength range. The RIN is calculated from 10 consecutive measurements, where a single measurement is an average of two-hundred spectra corresponding to a measurement time of 3.2 s and an average of a thousand pulses per spectral channel. The relative intensity variations between subsequent measurements can reach values as small as 1 $\%$ at the wavelength of 3 $\mu$m and increases to about 8 $\%$ at the wavelength of 3.5 $\mu$m. The dramatic variation in the 3.35–3.5 $\mu$m region, which coincides with the long wavelength edge of the SC spectrum, is attributed to the significant decrease in the power spectral density and large stochastic spectral power fluctuation of the SC source. These intensity fluctuations are typical characteristics at both the short and long wavelength edges of a supercontinuum spectrum^27^.

**Figure 4:**
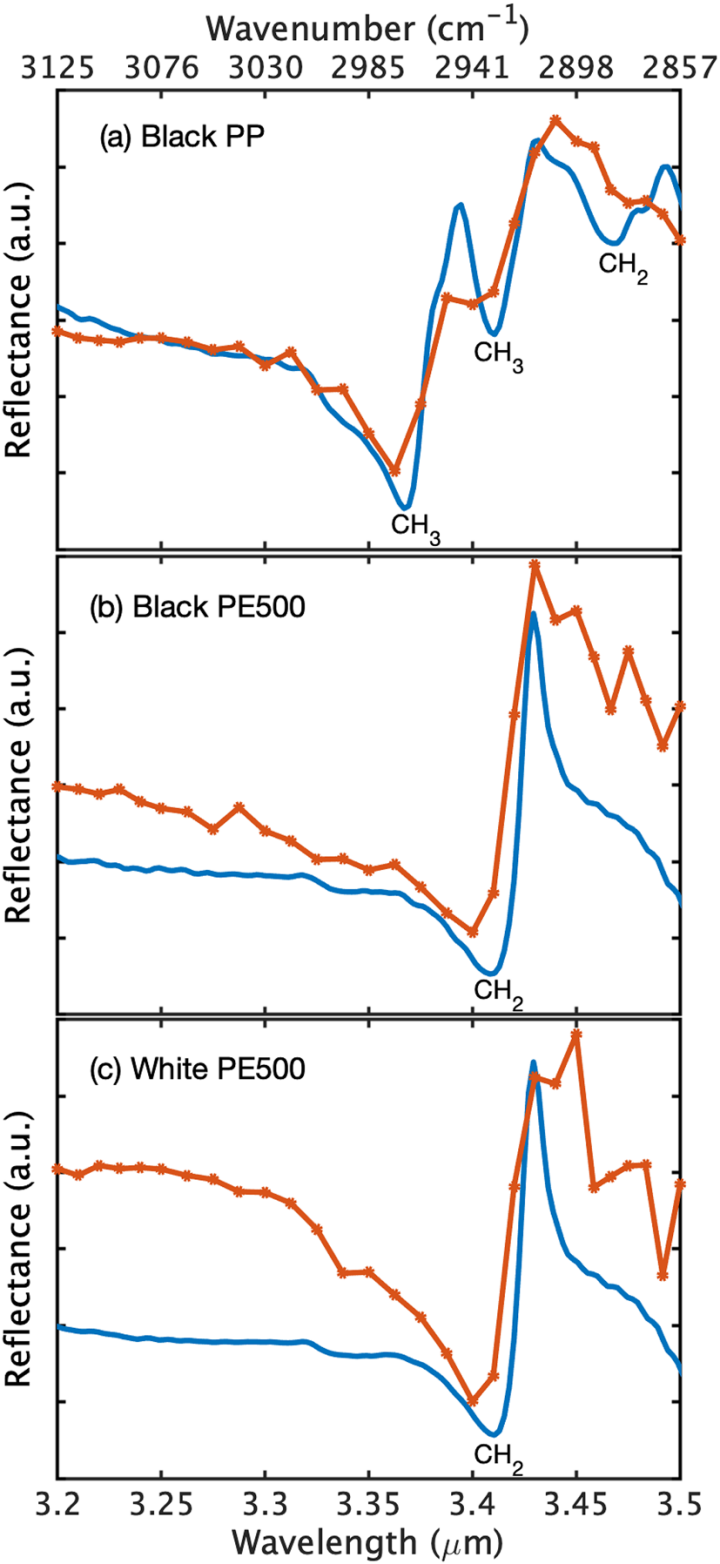
(**a**), (**b**) and (**c**) compares the reflectance spectra of black polypropylene (PP), black polyethylene (PE500) and white polyethylene (PE500), respectively. Solid blue line represents the spectra measured with FTIR spectrometer and the red line with a marker represents that of the active hyperspectral sensor (AHS).

A black polypropylene (PP) sample is investigated using the developed active hyperspectral sensor (AHS), and its corresponding reflectance is obtained using Eq. 1. The resulted spectrum is compared to a reference black PP reflectance spectrum measured with an FTIR spectrometer (FT-MIR Rocket, ARCoptix) having a spectral coverage of 2–6 $\mu$m and a resolution of 4 cm$^{-1}$. The comparison of the black PP reflectance spectra, measured with both the AHS and the FTIR spectrometer, is shown in Fig. 4a. The FTIR and AHS spectra demonstrate a very close agreement. The two reflectance peaks in the FTIR spectra between 3.3 and 3.45 $\mu$m are ascribed to the CH$_3$ functional groups while the peak between 3.45–3.5 $\mu$m is due to the CH$_2$ functional group^28^. Note that the AHS reflectance peaks are slightly broader in comparison to that of the FTIR. This is due to the relatively low spectral resolution of the FPI. Similarly, the FTIR and AHS reflectance spectra of black PE500 are compared in Fig. 4b. Both spectra are in excellent agreement as shown in the figure. The reflectance peak between 3.4–3.45 $\mu$m spectral region is assigned to the CH$_2$ functional group. Although both black PP and PE500 have somewhat similar spectral absorption features, their reflectance spectra are clearly distinguishable as shown in Fig. 4a, b respectively.

The AHS is further extended to the measurement of white plastics to ascertain the reliability of the technique as well as the impact of the additive coloring material. Fig. 4c presents a comparison between AHS and FTIR reflectance spectra of a white PE500. We can see a very good agreement between them. Moreover, the reflectance spectra of both the black and the white PE500 are very similar as can be seen in Fig. 4b,c, respectively. This is because the additive coloring material has negligible impact on the optical properties of the polymer at this wavelength range. This highlights the potential applicability of the sensor to differently colored plastics. There is a slight variation in the AHS spectra of the plastics. The cause for this is attributed to the fact that samples used in this work vary in surface quality (glossy, matte or rough surface), which leads to different light scattering properties. Nonetheless, all the absorption peaks are clearly present in the measured spectra and can be used for the identification and differentiation of the plastic species.

## Conclusion

We developed a novel hyperspectral sensor using a mid-infrared supercontinuum light source and a tunable MEMS-based Fabry-Pérot interferometer. The FPI enables robust wavelength selection across the probed spectral range of the SC spectrum. The FPI is engineered to be standalone, thereby enabling robust sensor design as positioning of the FPI on the sensor is flexible. For instance, placing the FPI in front of the laser would allow target illumination with only the desired spectral components with relatively low power compared to the whole SC spectrum, this is particularly important in applications where the target sample has very low damage threshold. Our preliminary demonstration of line filtering with a FPI of similar principal mechanism operating in the near infrared, with an aperture size of about 1.5 mm, showed a power handling of more than 10 W of continuous wave of a 1–2 $\mu$m SC laser. We demonstrated hyperspectral sensing of black polyethylene (PE500) and polypropylene (PP) using the 3–3.5 $\mu$m band of the SC spectrum. The measured reflectance spectra of the plastics are compared to their reference measured with an FTIR spectrometer. An excellent agreement was observed between the spectra. Furthermore, we measured the reflectance spectra of a white PE500 to emphasize the applicability of the sensor to differently colored plastic samples. Our results showed, for the first time, the suitability of the FPI for active hyperspectral identification of polymers. The sensor demonstrates a measurement rate of about sixty-five spectra per second, limited by the tuning frequency of the FPI, and a relative intensity noise of 1–8 $\%$ in the 3–3.5 $\mu$m wavelength range. The main sources of noise in this work are the supercontinuum light source spectrum instabilities and the readout noise associated with the detector, amplifier as well as the FPGA. It is important to emphasize that the measurement speed and accuracy can be significantly improved by optimizing the 0.5 duty cycle scanning frequency of the Fabry-Pérot interferometer to make use of the full cycle, which would double the spectrum acquisition rate and enhance the signal level during the same acquisition time, and thus increase the signal-to-noise ratio by a factor of $\sqrt{2}$. Further improvement can be achieved by tailoring temporal scanning profile so that noisy part of the spectrum would have longer integration time compared to other parts of the spectrum. Furthermore, the biggest improvement can be realized by using better tailored supercontinuum light source where the wavelength range of interest is not located at the far edge of the SC spectrum. This would result in increase of average spectral density in that region, and significantly decrease shot to shot fluctuations thereby enhancing the signal-to-noise ratio of measured spectra. The aforementioned optimizations would enable real-time plastic wastes sorting and other hyperspectral sensing applications in the mid-infrared.

## Data Availability

The datasets used and/or analysed during the current study are available from the corresponding author upon reasonable request.
